# 

**Published:** 1898-07-16

**Authors:** 


					THE HOSPITAL. "The standard of highest purity."?Lancet, .Jnr.v 16tit, 1S9S.
CADBURY'S w CADBURY'S
COCOA
m
ABSOLUTELY PURE. Jgb *=? - NO DRUGS OR ALKALIES USED.
COCOA
Hnspt T& L
No. 6I;6. Vol. XXIV. HIS!] Saturday
July 16th, 1898.
[SuhaariptionTem^ pRICE TWOPENCE.

				

